# Activity-Free User Identification Using Wearables Based on Vision Techniques

**DOI:** 10.3390/s22197368

**Published:** 2022-09-28

**Authors:** Alejandro Sanchez Guinea, Simon Heinrich, Max Mühlhäuser

**Affiliations:** Department of Computer Science, Technical University of Darmstadt, 64289 Darmstadt, Germany

**Keywords:** user identification, image representation, CNNs, IMU, inertial sensors, wearable sensors

## Abstract

In order to achieve the promise of smart spaces where the environment acts to fulfill the needs of users in an unobtrusive and personalized manner, it is necessary to provide means for a seamless and continuous identification of users to know who indeed is interacting with the system and to whom the smart services are to be provided. In this paper, we propose a new approach capable of performing activity-free identification of users based on hand and arm motion patterns obtained from an wrist-worn inertial measurement unit (IMU). Our approach is not constrained to particular types of movements, gestures, or activities, thus, allowing users to perform freely and unconstrained their daily routine while the user identification takes place. We evaluate our approach based on IMU data collected from 23 people performing their daily routines unconstrained. Our results indicate that our approach is able to perform activity-free user identification with an accuracy of 0.9485 for 23 users without requiring any direct input or specific action from users. Furthermore, our evaluation provides evidence regarding the robustness of our approach in various different configurations.

## 1. Introduction

Providing support to everyday life while disappearing into the background has long been seen as the goal of ubiquitous computing [1]. An essential task towards this goal is the ability of the system to provide personalized services, for which it is necessary to recognize the user interacting with the system at any given time. This is known as *user identification* [2]. The idea of user identification is to match a newly obtained biometric sample from an already registered user against all records in the system’s existing database and to then indicate to which of the registered users that sample corresponds [3]. We can say that, for user identification to adhere to the goal of ubiquitous computing, it needs to minimize intrusiveness and avoid demanding any kind of direct input or specific action from users that may interrupt the regular flow of their daily routines [4].

Many different user-identification approaches have been proposed in the past. A large body of work has been performed around biometrics, such as fingerprints, irises, and faces; however, while accurate, they all require direct input from the user [5]. Approaches that seek to alleviate this issue are based on the behavior patterns of users. In this respect, however, most approaches have focused on performing identification based on a small set of gestures, actions, or activities. Some examples include approaches based on gait patterns (e.g., [6,7]), hand-waving gestures (e.g., [8]), and based on multiple activities, such as sitting, standing, and walking [9].

The dependence of this type of methods on specific gestures, actions, or activities may be seen as representing an interruption on the regular flow of the daily routines of users. Recently, some approaches that solve activity recognition and user identification simultaneously have been proposed [5,10], thereby, making the identification more flexible in terms of the activities that may be considered. However, those methods are, at their core, activity-based approaches, which use learned activity information to perform the identification. This necessarily constrains the identification to operate based on a specific set of activities.

To the best of our knowledge, there is only one previous work that has proposed an approach that attempts to free the user from any gestures, actions, or activities while performing the identification, under the term continuous user identification [11]. In that work, the authors presented an approach that, using wavelet transform over quaternions obtained from a wrist-worn IMU, performed identification without requiring any direct input or specific action from the users. Despite this, the approach suffered from low accuracy, which was particularly noticeable as the number of people considered in the identification increased.

In this paper, we propose an activity-free user-identification approach (see Section 3), based on data recorded by a wrist-worn Inertial Measurement Unit (IMU). Our approach is able to perform the identification at any given point in time without requiring any direct input or specific action from users to operate, and instead it is capable of performing the identification at any point in time throughout the regular daily routine of users.

Furthermore, our approach is based on a wearable, since we believe that it is an option that may better suits users’ privacy concerns, since they can decide to remove it and thus stop the recording at will, which is not possible with device-free approaches, based on, for instance, WiFi signals or video recording. A key aspect of our approach is the representation of IMU time-series data as images, which has proven relevant for the task of activity recognition [12] and has shown promising results in the past for user identification [13]. This method allows to exploit current vision techniques to achieve high levels of accuracy and robustness without having to deal with overly complex machine-learning models.

To asses the performance of our approach, we conduct an extensive evaluation that considers different relevant configurations, including different specific image representation designs, different numbers of registered users, different training dataset sizes, and different convolutional neural network architectures (see Section 4). According to our results (see Section 5), the accuracy of our approach under the best configuration was 0.9485 for 23 registered users. Furthermore, our results provide evidence on the robustness of our approach to various different configurations.

The main contributions of our work are summarized as follows:

- An activity-free user identification that does not require any direct input or specific action from users (see Section 3.1).
- A further exploration of the use of image representations of IMU data to obtain high performance levels based on vision techniques, which has proven relevant in the past for activity recognition [12] and has shown promising results for identification [13]. We show that the image representation that we propose improves the performance of CNNs compared to their application directly on raw time-series data (see Section 3.2)
- An extensive evaluation that demonstrates the effectiveness and robustness of our approach and provides insights that may be helpful both for practitioners interested in implementing our approach in real-world applications as well as for researchers seeking for future lines of work.

The remainder of this paper is organized as follows. In Section 2, we review the related literature. Our approach is fully described in Section 3, including the whole pipeline and the image representation that we propose. The setup, rationale, and detailed description of our experiments are in Section 4. Then, in Section 5, we provide our results and analysis. Finally, in Section 6, we provide our conclusions, including some potential lines for future work.

## 2. Related Work

### 2.1. Motion-Based Identification

#### 2.1.1. Single and Multiple Gesture/Action/Activity-Based Identification

A large body of work on motion-based user identification has considered walking as the activity from which the identification is to be performed. For instance, in [6], the authors presented an approach that profiles human movement using WiFi signals and, based on that, performs the identification. A similar device-free, indoor identification based on gait patterns was presented in [14].

In [7], the authors presented an approach based on deep learning that identifies people based on their gait patterns obtained from inertial sensors in smartphones. With respect to gestures, hand waving is a gesture that has been widely considered for user identification (e.g., [8]).

Seeking to more broadly encompass a user’s daily activities, different approaches have considered multiple gestures, actions, or activities to perform identification. Examples of multi-gesture identification approaches are an approach that uses head-mounted displays and considers blinking and head movements to perform the identification [15], an approach based on WiFi as sensing method and gestures, such as kicking and waving [16], and an approach considering various gestures (e.g., hand waving, come-over, one-hand raised, and phone-to-ear), which are performed either standing or sitting and recorded by a Microsoft Kinect [8].

In terms of multi-action/activity approaches, we have examples, such as an approach that considers walking, jogging, and going up/down stairs for identification [2], an approach that considers talking and stationary activities (e.g., working in front of the computer and operating a hot stove) [17], and an approach that considers, among others, household cleaning activities and office-work activities [18].

Recently, multi-activity-based approaches that are more flexible in terms of the set of activities considered have been proposed (e.g., [5,10]). These approaches are focused on solving activity recognition and user identification simultaneously, transferring knowledge between these tasks to improve jointly the performance of both. In [10], the authors proposed a deep-learning approach in which two deep neural networks (one for activity recognition and one for user identification) were combined to solve both tasks. In [5], the authors proposed an approach based on a deep-learning siamese neural network and temporal convolutions that allows solving both activity recognition and user identification with no explicit labeling information given.

The main difference between gesture/action/activity-based approaches and our activity-free identification is that, in our case, the approach does not depend, require, or expect any specific action from the user to perform the identification. This is also true if we consider approaches that allow users to perform any of a set of multiple gestures, actions, or activities, since no matter how comprehensive the set is, it remains hard to encompass everything that is entailed in the daily routines of users.

The more recent developments that simultaneously solve activity recognition and activity-based identification (e.g., [5,10]) provide more flexibility about the type of activities that may be considered. However, the models they propose are based on sharing information learned about the activities to perform the identification. In this respect, our approach does not use any activity-related information, and thus performs the identification giving complete freedom to the users to follow their daily routines without any restrictions.

#### 2.1.2. Activity-Free Identification

A number of approaches in the past have claimed to be focused on activity-free user identification. However, most of them are in fact either multi-modal or based on multiple gestures, actions, or activities. Among these approaches, we have an approach based on the people’s bodies shape as captured by an array of cameras while the people perform activities, such as eating and drinking, waving, walking, and running [19]. Another example approach, also claiming to perform continuous identification, considers keystroke and mouse motion patterns for identification [20].

To the best of our knowledge, only the work in [11] has truly focused on activity-free identification, although this is referred to as continuous identification. That approach proposes the use of wavelet transform over quaternions that are computed from the inertial sensor data recorded by a wrist-worn IMU. The main issue with the work presented in [11] is the accuracy of the approach, which, even for the smallest group of registered users (i.e., two users), remained around 0.88.

Moreover, as the size of the group of registered users became larger, the drop in accuracy became particularly pronounced, going down to as little as 0.63 for the case of 10 registered users. In this respect, our approach improves considerably upon the performance of this existing work, both in terms of the accuracy and robustness to changes in the number of registered users (acc = 0.9487 for 23 users).

### 2.2. CNNs for Time Series

Some previous works have used CNNs as part of their user-identification approaches (e.g., [5,10,21]). Similar to [22], the approaches in [10,21] combined CNNs with LSTMs. The approach in [21] used a CNN to obtain high-level features that are related to user behavior and physical characteristics. This is similar to the way the approach in [10] used CNN for automated feature engineering. In [5], the authors used a distinct type of convolutional network, specifically a temporal convolutional network (TCN), which, through a hierarchy of convolutions, is able to entail the temporal relations of the time-series data at different time scales. In contrast to these methods, our approach focuses on obtaining an image representation with as much relevant information as possible to allow even a simple CNN to yield good results.

### 2.3. Image Representations of Time Series

The main differences between our approach and other existing approaches that either use images that depict directly the input signals on images (e.g., [23,24]) or those that apply specific encoding, such as Gramian Angular Field (GAF) and the Markov Transition Field (MTF) (e.g., [25,26]) is that, in our case, we focus on producing image representations that represent as many patterns as necessary in a way that takes specific advantage of the strengths that have been long recognized in CNNs [27].

## 3. Approach

### 3.1. Identification Pipeline

The user identification that we propose in this paper follows a pipeline that is inspired by the pipeline presented in [11], where the identification was obtained in two steps. First a machine-learning classification is performed over the instances of the input time series, where each instance corresponds to non-overlapping windows. Then, the final identification is produced based on a voting mechanism among predefined decision segments that contain a number of windows of the time series. The key difference in our case is the image representation process, which takes the time-series data and transforms it into images that are then fed into the machine-learning-classification method. As a classification method, we consider a convolutional neural network (CNN), since CNNs have proven to be particularly well-suited in dealing with image data [28].

**Figure 1:**
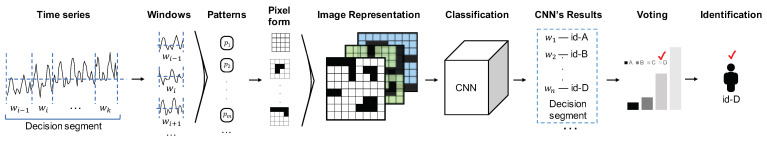
Pipeline of our approach.

The pipeline of our approach is depicted in Figure 1. On the left-hand side, we can see the time-series data as received from the IMU (only depicting a single channel for simplicity; however, we have one time series for each *x*, *y*, *z* component of each of the three inertial sensors in the IMU, i.e., accelerometer, gyroscope, and magnetometer). The time series is divided into non-overlapping windows of same size. We decided to use non-overlapping windows. We attempted different configurations for the sliding window mechanism, and we noticed that there was no significant difference in accuracy when considering overlapping windows; however, overlapping considerably increased the computation time.

Following the identification pipeline illustrated in Figure 1, different patterns related to the variation over time of the sensor data are computed for each of the windows in the time series (see Section 3.2.2). The patterns corresponding to a single window are then represented in pixel form according to predefined filling strategies (see Section 3.2.4), and the resulting pixel regions are placed altogether into an image representation following a predefined layout (see Section 3.2.3). This is repeated for the whole time series to obtain one image representation for every window.

We consider the possibility of generating either black and white (B&W) or color (RGB) images. Once the image representations are generated, they serve as the input instances of a CNN that classifies each to a particular identity class. The next step is to use the results from the CNN and consider a voting round among the classified instances of *decision segments* that encompass a predefined number of windows. The voting mechanism yields the final identification based on the identity class that according to a predefined criteria is considered the winner within each decision segment.

### 3.2. Image Representations

#### 3.2.1. Intuition and Rationale

The intuition behind the use of image representations of time-series data for IMU-based user identification is to take advantage of convolutional neural networks (CNNs), which have shown the best results when used on image data [28]. In this way, we aim at boosting the performance of the identification, inspired by the promising results previously obtained in [13].

The image representations we propose are designed with the following objectives in mind: (a) representations that are particularly well suited for motion-based user identification, (b) representations that allow to include as many patterns that are relevant to distinguish individuals by their arm and hand motions, and (c) representations that play to the strengths of CNNs, particularly locality and edge detection [27].

In order to address (a), we do not represent time-series data directly (as some previous works have suggested (see Section 2.3)); however, we instead consider patterns that describe the variation over time in the data obtained from the inertial sensors. Concerning (b), we consider extending the designs of image representations that have been previously proposed to allow for the inclusion of as many relevant patterns as needed [12,13]. Finally, in what relates to (c), we define the representations as a compound of consistent regions that are filled with pixels in a way that represent the pattern values as continuous lines.

#### 3.2.2. Patterns

**Figure 2:**
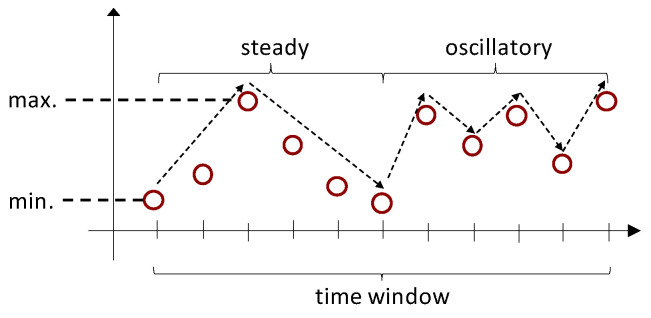
Variation patterns over time.

We focus only in representing patterns over time. Specifically, for each of the time-series components we consider the oscillatory and steady variations and the range of values over a time window of predefined size. The oscillatory variation is seen as such in which the value of the time series increases and decreases in every immediate subsequent time step. The steady variation refers to the case in which the value of the time series either only increases or decreases over a period of time. The change in value in both types of variation can be of any magnitude. Concerning the range, we consider the maximum and the gap between maximum and minimum values. Figure 2 illustrates these patterns.

For the computation of the variation patterns, the window is divided into consecutive non-overlapping segments, according to the type of variation. Each segment spans not less than three data points in order to be able to distinguish if the transitions between data point values indicates a steady or a oscillatory variation. The value of the variation *v* is then computed for each of the segments, according to the formula in Equation (1), where $d_{i}$ corresponds to a value of a data point at step *i* and $d_{i-1}$ to the value at the previous step.

(1) $$v=\frac{1}{k-1}\sum_{i=1}^{k}d_{i}-d_{i-1}$$

Finally, the value for each type of variation is obtained by averaging the values of all segments of the same type of variation within the current window.

#### 3.2.3. Image

**Figure 3:**
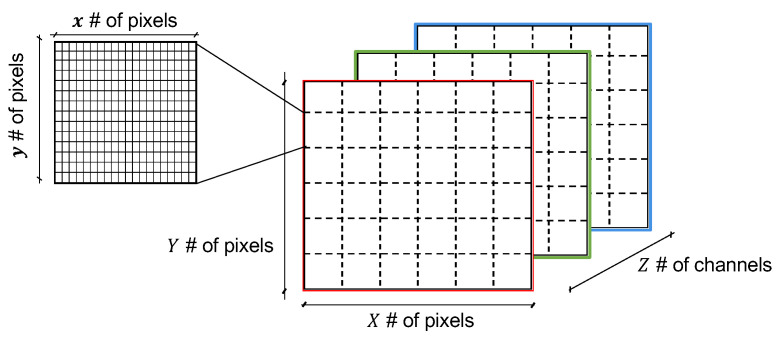
Image and regions.

For each window in the time series, we generate one raster image or bitmap [29], either black and white (B&W) or color (RGB). Our goal is to combine the patterns values found within a window into an image. For that purpose, we define an empty bitmap of X×Y number of pixels and *Z* number of channels, divided into several regions of x×y number of pixels, where each region is meant to represent one pattern value. This is illustrated in Figure 3. For this paper, we consider for the single-channel (B&W) case, an image divided into 36 (6×6) regions, where *x* and *y* are the same for all regions.

#### 3.2.4. Patterns to Pixels

To define the correspondence between a pattern value and the pixels in a region, we first consider pixels as marked or unmarked, where a marked pixel has a value of 255 (i.e., a white pixel) and an unmarked pixel has a value of 0 (i.e., a black pixel). To obtain the number of marked pixels corresponding to a given pattern value, we define a mapping *M* between the minimum ($\min_{p}$) and maximum ($\max_{p}$) values of the given pattern throughout the whole time-series data and the minimum ($\min_{px}$) and maximum ($\max_{px}$) number of pixels that can potentially be marked within a region.

This allows to transform any given pattern value *x* into the corresponding number of marked pixels within a region according to Equation (2).

(2) $$M\left(x\right)=\left\lfloor \min_{px}+\left(\frac{\max_{p}-\min_{p}}{\max_{px}-\min_{px}}\right)\left(x-\min_{p}\right)\right\rfloor$$

**Figure 4:**
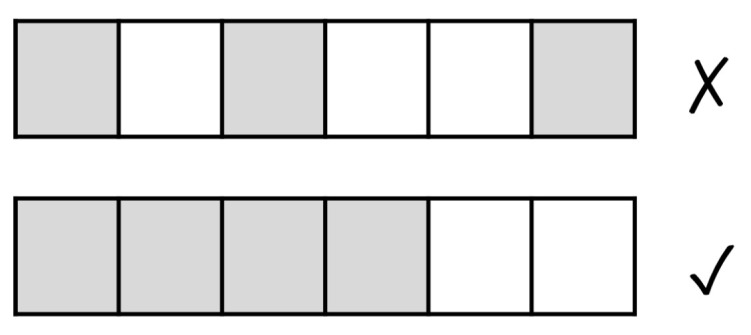
Continuous pixel marking (edge detection).

The value of M(x) is ensured to be a whole number (through the use of the floor function) to make the translation into number of marked pixels easier. The pixel marking is performed as to mark contiguous pixels according to a predefined path. This is illustrated in Figure 4.

### 3.3. Classification

For the classification part of our approach, we follow two steps. First, we use a convolutional neural network (CNN) to obtain a tentative class for each example in the data that corresponds to a window within the time series. Then, we define *decision segments*, which divide the time series into chunks of the same number of windows. Finally, we apply a voting mechanism, according to a predefined criteria, to decide the class for each of the decision segments and consequently for the examples within.

The voting mechanism in the second step provides two advantages. First, it obtains further information from patterns occurring close in time to take the decision. Second, it allows the possibility for a second opinion, if it is not possible to decide on one specific class, instead of going directly to a misclassification. Our approach is not concerned with the specific way in which a second opinion is taken; however, instead, it leaves such cases as *undecided* or a reject.

**Figure 5:**
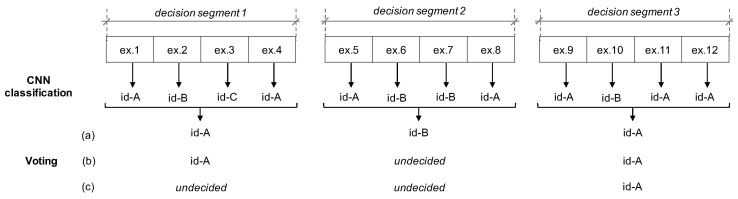
Example of the voting mechanism. Three different voting criteria are represented in this example: (**a**) first to plurality, (**b**) plurality, and (**c**) majority.

We consider four possible voting criteria, of which only one is used in the execution of the approach: first to plurality, plurality, win by *x* number of votes, and majority. Majority and plurality are typical voting strategies [30]. In majority voting, the winner must have more than 50% of the votes. In contrast, plurality gives the win to any with at least one vote more than the rest. The criterion first to plurality is meant to consider a case where the number of undecided is removed entirely, since even in the case of a draw the winner is the first class to have obtained more votes. In Figure 5, we show a simple example that illustrates the two-step classification, particularly on the different classifications that the voting may yield depending on the specific criterion used.

## 4. Evaluation Methodology

### 4.1. Experimental Setup

#### 4.1.1. Dataset

We collected data from 23 people between 25 and 35 years old (mean: 29.13; std: 3.09). The collection took place throughout 2021 and beginning of 2022. All the participants were researchers working at the university. The environment where the participants went about their regular routines is an office space inside a university building. The participants were asked to follow their daily office routines and no specific gesture, action, or activity was required from them.

We considered an office, computer-science, research-like environment, where we collected, for each participant, data over three sessions, each recorded on a different day. The duration of each session was of approximately 5 h. The IMU used for collection was the Shimmer3 [31], calibrated at a sampling rate of 51 Hz. The data includes the *x*, *y*, *z* components of each of the inertial sensors in the IMU, i.e., accelerometer, gyroscope, and magnetometer. We plan to make the data fully available in a public repository in the near future. The data collection was conducted in accordance to the guidelines of the ethics commission of our university.

The reason for collecting our own dataset, instead of using for our evaluation an existing activity-based dataset, is to avoid what we see as a direct threat to the validity of our evaluation. Although we could disregard the activity labels, any existing activity-based dataset contains data collected from people performing a specific set of activities, which in our view, no matter how comprehensive this set may be, constrains a daily routine to only some foreseen activities, which might not be realistic in many cases. Therefore, in seeking a sound evaluation, we decided to consider a dataset collected from people that perform their daily routines unconstrained without any direct input or specific action from their side.

In both our preliminary experiment (Section 4.2) and our main experiments (Section 4.3), we considered a dataset that we collected, following a setup similar to the one in [11]. We did not use the dataset provided in [11] because their dataset does not provide the raw data as recorded by the inertial sensors in the IMU but only includes the quaternions computed from the inertial sensor raw data. This is a key factor for us because, as part of our evaluation, we wanted to examine if the raw data could have an impact on performance.

#### 4.1.2. Basic Experimental Configuration

The basic configuration of our approach for the evaluation consists of a windows size of 51 data points (i.e., 1 s of data) and decision segments that span 30 windows (i.e., 30 s of data). For all but Experiment 2, which compares the performance of our approach for different voting criteria, we consider first to plurality, since this criterion allows us to have no undecided decision segments.

In all our experiments, except for Experiment 4 (see Section 4.3.4), we use a convolutional neural network (CNN) defined as follows. Two convolutional layers, each considering 128 filters of size 3×3. Each convolutional layer is followed by a max-pooling layer, with filter size of 2×2 and a stride of 2. These layers are followed by a dense layer of 256 units. In all cases, we obtain our results considering 10-fold cross-validation.

#### 4.1.3. Evaluation Metrics

The main metric that we use in our evaluation is *accuracy*, defined as the number of instances that are correctly classified to the right identity divided by the total number of instances.

### 4.2. Preliminary Experiment

Our preliminary experiment is meant to help us find the appropriate baseline, against which the performance of our approach is to be compared. We consider as baseline the approach presented in [11], which is the only previous work that targets a truly continuous identification. However, there are two aspects that we believe are relevant for our particular case:

*(a)*  Using CNN over the time-series data directly, instead of the *k*-NN and Random Forest (RF) techniques considered in [11]. We considered this aspect, since our approach is based on the use of a CNN over image representations of the time-series data. Therefore, it is relevant to observe the performance of the approach when considering the same CNN working on the time-series raw data directly.
*(b)*  Establishing the best possible type of time-series data to be used as input. Starting with the wavelet transform (WT) over quaternions as used in [11], passing through the use of quaternions directly without transformation, and finishing up at the raw level, considering directly the data from the inertial sensors in the IMU (i.e., accelerometer, gyroscope, and magnetometer), which are used for the computation of quaternions, we did not consider the other well-known approach for analyzing the frequency content of time-series data—namely, Fourier transform—since previous works have indicated that Fourier transform introduces resolution issues, particularly when dealing with sudden changes of frequency throughout time [32].

In order to address the aspects above, we design an experiment in which we compare the performance of the approach presented in [11] using a CNN (instead of RF), with, on the one hand, quaternions as input and, on the other hand, the raw data directly taken from the inertial sensors in the IMU. For this preliminary experiment, we considered a reduced number of participants (*N* = 10) from the dataset.

### 4.3. Main Experiments

#### 4.3.1. Experiment-1—Design Comparison

In this experiment, we evaluate a number of specific image representation designs that follow the basic general design elements of our approach, presented in Section 3.2. For each design, we measure the average time (in seconds) that an image representation takes to be generated (i.e., the average generation time) and the accuracy of the model trained using that design. To measure the average generation time, we consider the images corresponding to the whole dataset. Concerning the measurement of the accuracy, we consider only the case of the maximum number of registered users that we have—namely, 23 users.

**Figure 6:**
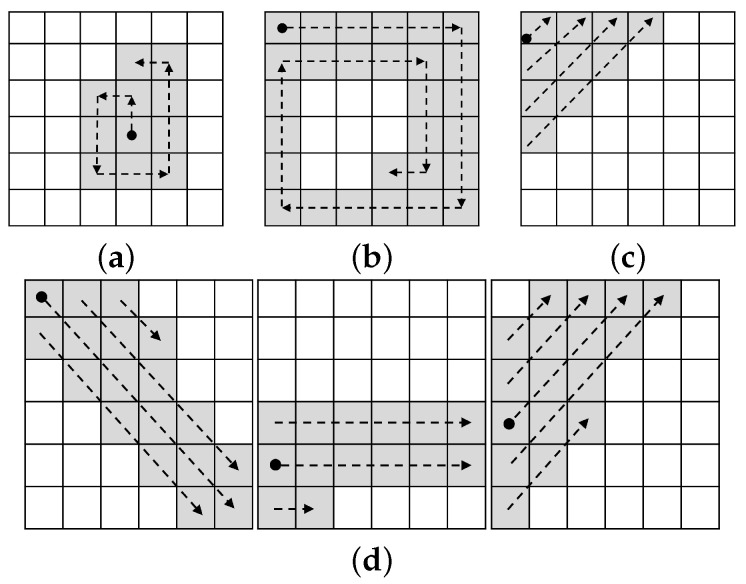
Filling strategies. (**a**) Counterclockwise, (**b**) Clockwise, (**c**) Diagonal, and (**d**) Strokes.

We consider four different filling strategies for our evaluation. These strategies refer to different ways in which to mark contiguous pixels according to a predefined path. The strategies that we consider are aimed at producing images with well-localized and distinguishable edges that can be easily reproduced. Next, we describe each of the filling strategies that we consider, which are illustrated in Figure 6.

*Counterclockwise (CCW)*  This strategy is depicted in Figure 6a. The idea is to mark the pixels consecutively starting on the middle of the region and following a counterclockwise path, without passing over the same pixel twice.
*Clockwise (CW)*  This strategy (depicted in Figure 6b) follows a continuous clockwise path, starting on the top left, leaving no pixel unmarked, and never passing through the same pixel more than once.
*Diagonal (Diag)*  The diagonal strategy (as seen in Figure 6c) is based on 45° diagonal that are drawn upwards, starting at the pixel of the top-left corner, leaving no pixel unmarked in a non-overlapping manner.
*Strokes (Strk)*  This strategy is meant to yield continuous strokes across three different regions on the same row of the image, as illustrated in Figure 6d. From left to right, the first region is filled using reverse diagonals starting on the top-left corner, the next region is filled using horizontal lines that start on the row where the previous region ended, and the last region is filled using diagonals starting on the row where the previous region ended.

We consider two general designs for the layout of the regions in the canvas: design A and design B. The main difference between these designs is on how the patterns are laid down across the regions and the channels of the canvas.

**Figure 7:**
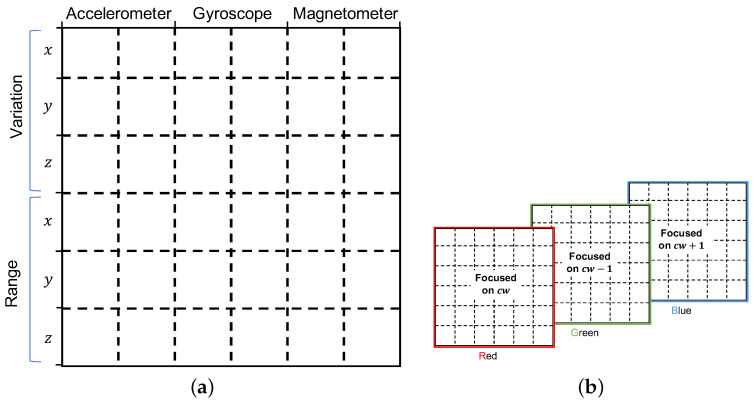
Image representation—Design-A. (**a**) Single channel and (**b**) Multi-channel.

*Design A.* In this design, the patterns for the current window of all sensors are represented in a single channel. This is illustrated in Figure 7a, where the variation patterns and the range of the the three components (*x*, *y*, and *z*) from the accelerometer, the gyroscope, and the magnetometer are all represented in a single channel. In the RGB case, the two extra channels are used to represent the same patterns but for the preceding and following windows as can be seen in Figure 7b.

**Figure 8:**
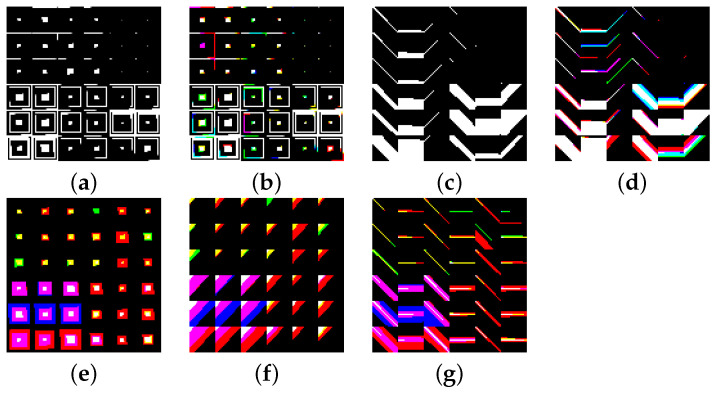
Samples of the image representation designs. (**a**) A-1, (**b**) A-2, (**c**) A-3, (**d**) A-4, (**e**) B-1, (**f**) B-2, and (**g**) B-3.

We evaluate four different variations of Design-A as follows:

*Design-A-1*  A single channel is considered in this design (B&W images). The patterns of the current window for the accelerometer, gyroscope, and magnetometer are, respectively, represented on the first, third, and fifth region, counting from left to right. The second, fourth, and sixth regions are, respectively, representing an extended window that considers the current window and the ones before and after, for each of the sensors in the IMU. Furthermore, each region is filled using both counterclockwise and clockwise strategies. Each region assigned to the *variation* patterns represents both oscillatory and steady variations, using the counterclockwise strategy for the oscillatory and the clockwise strategy for the steady variation.
  This is made without allowing overlapping, for which we restrict $\max_{px}$ to 200 for each pattern. Concerning the regions that represent the range, we consider three different values: the maximum, the difference, and the mean. The clockwise strategy is used for the maximum and the mean, while the counterclockwise strategy is used for the difference. No overlapping is allowed, for which we restrict $\max_{px}$ to 144 for the maximum and the difference patterns, and to 112 for the mean. The mean pattern starts at the 145th pixel. An instance of this design can be seen in Figure 8a.
*Design-A-2*  This design is the RGB version of *Design-A-1*. A sample of this design is depicted in Figure 8b. As in any RGB version of *Design-A*, the red channel is focused on the actual current window, while the green channel focuses on the previous window and the blue channel on the next window.
*Design-A-3*  This design uses strokes as the filling strategy and only the current time window from the data. On the top of the bitmap, the first, third, and fifth columns represent the corresponding oscillatory variation, and the representation of the maximum range is placed on the bottom. The second, fourth, and sixth columns represent the corresponding steady variation on the top of image, and the difference between maximum and minimum on the bottom. A sample of the images produced using this design is depicted in Figure 8c.
*Design-A-4*  This design is the RGB version of *Design-A-3* (a sample appears in Figure 8d).

**Figure 9:**
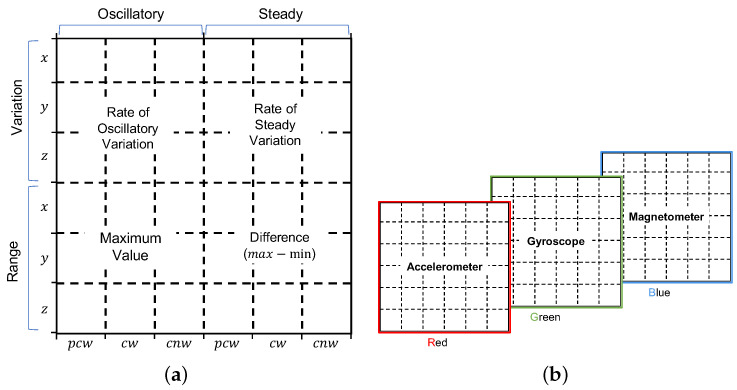
Image representation—Design-B. (**a**) Single channel and (**b**) Multi-channel.

*Design B.*Figure 9 depicts the layout of design B. As can be seen in the figure, each channel represents the patterns from one of the sensors in the IMU. In this case, the B&W case (in Figure 9a) only represents patterns from the accelerometer, and in the RGB case (in Figure 9b), the gyroscope and the magnetometer are represented in the green and blue channels, respectively.

As it is shown in Figure 9a, the bitmap for each channel can be seen as divided into four main super-regions of nine regions each, where the top ones are meant to represent the variation in the three components (*x*, *y*, *z*) of the corresponding sensor data, and the bottom two are dedicated to the range, one for the maximum value and one for the difference between maximum and minimum. Each of these super-regions consider the current window (*cw*) and two extended windows, one spanning the preceding and current windows (*pcw*), and one spanning the current and next windows (*cnw*).

Specifically, we evaluate three different variations of Design-B, all of which are RGB and vary between each other only on the filling strategy considered: *Design-B-1* (counterclockwise), *Design-B-2* (diagonal), and *Design-B-3* (strokes). A sample of each of these designs is depicted in Figure 8e, Figure 8f, and Figure 8g.

#### 4.3.2. Experiment-2—Voting Criteria Comparison

In this experiment, our goal is to evaluate, depending on the specific voting criteria considered, how much of the error is undecided (or rejected) and how much is misclassified. This comparison is relevant if we consider the case in which, given an undecided identification, which we consider a reject, it would be possible to use a supporting approach to take the final decision, instead of obtaining the misidentification directly.

We use the following metrics for this experiment. *True Acceptance Rate*, which is defined as the number of instances that are accepted (or not undecided) and correctly classified to the right identity divided by the total number of instances. In our case, it corresponds directly to the accuracy. *Misclassification Rate*, defined as the number of instances that are incorrectly classified to the wrong identity divided by the total number of instances. Finally, *False Rejection Rate* which is defined as the number of instances that are rejected (or undecided) divided by the total number of instances.

We consider six different voting criteria, which are explained in Section 3.3: first to plurality, plurality, win by three votes, win by six votes, win by nine votes, and majority. For this experiment, we use the design that obtained the best accuracy in the benchmarking of Experiment 1.1.

#### 4.3.3. Experiment-3—Training Size

In this experiment, we focus on varying the size of the training dataset. The intent is to measure the impact that different sizes of the data used for training may have on the performance of our approach. The rationale is that in real-world applications the users will expect the system to require a minimum training period before operation. The sizes we consider for the training dataset are 5 (the typical size of a session of collected data per user), 3, 2, 1, and 0.5 h. In all cases, the testing set is formed by the rest of the collected data.

For each of these cases, the ratio of the size of the training dataset with respect to the size of the whole data is approximately 1/3 (i.e., 5 h for training and 10 h for testing), 1/5 (i.e., 3 h for training and 12 h for testing), 2/15 (i.e., 2 h for training and 13 h for testing), 1/15 (i.e., 1 h for training and 14 h for testing), and 1/30 (i.e., 0.5 h for training and 14.5 h for testing), respectively. In either case, the data splits are formed by taking at random samples from all the three sessions of collected data.

In this experiment, we consider all the possible number of registered users, from 2 to 23. For all but 23 registered users, we consider five different combinations of users in order to account for the variation of the performance of our approach depending on the specific group of people considered as registered users in the identification. This criteria follows the method proposed by [33] to for a more rigorous evaluation of identification approaches.

#### 4.3.4. Experiment-4—CNN Architectures

We evaluate the performance of our approach under four different CNN architectures. This is meant to show how the performance may be impacted by the design of the architecture. In all cases, we consider simple architectures, with some being deeper and/or wider than others.

The specific architectures that we consider are as follows:

*CNN-0*  This architecture consists of two convolutional layers, each considering 128 filters of size 3×3. Each convolutional layer is followed by a max-pooling layer, with filter size of 2×2 and a stride of 2. These layers are followed by a dense layer of 256 units. (This is the architecture used throughout all our evaluation)
*CNN-1*  This architecture is defined first with a convolutional layer using 16 filters of size 3×3 and a stride of 2, then a max-pool layer with filter size of 3×3 and a stride of 2, followed by a convolutional layer using 32 filters of size 3×3 and a stride of 1, then again the same max-pool layer as before, and finally a dense layer with 120 units.
*CNN-2*  This architecture considers three different convolutional layers, the first one using 16 filters of size 3×3 and a stride of 1, the second one using 32 filters of size 3×3 and a stride of 1, and the third one using 64 filters of size 3×3 and a stride of 1. Each of these layers is followed by a max-pool layer with filter size of 3×3 and a stride of 2. At the end, one dense layer with 256 units is placed followed by another dense layer with of 120 units.
*CNN-3*  This architecture is defined as follows. First two convolutional layers using 32 filters of size 3×3 and a stride of 1, each followed by a max-pool layer with filter size of 3×3 and a stride of 2. This is then followed by a convolutional layer using 64 filters of size 3×3 and a stride of 1 and again the same max-pool right after. Two dense layers finish the architecture, first one with 256 units and then one of 120 units.

The design of architecture *CNN-0* could be considered as naive, since although it follows the traditional construction of a CNN, in terms of types of layers and their order, it defines convolutional layers with a large number of filters and of exactly the same size, both of which are typically not recommended [34]. The rest of the architectures are more in accordance with the construction that is widely used for CNNs, with the size of the layers increasing as they are deeper. In fact, we use *CNN-0* throughout most of our evaluation, since we aimed to show that our approach is robust even to not-so-ideally designed CNN architectures.

### 4.4. Experiment-5—Performance for Different Numbers of Registered Users

In this experiment, we evaluate the difference in performance depending on the number of users registered in the system, as this is a known issue of identification approaches [11]. We evaluate our approach for as few as two registered users and all the way until 23 users to observe the difference in the performance throughout. The results of this experiment are obtained using 10-fold cross-validation.

Furthermore, we consider five different combinations of users out of the total number of participants for each of the number of registered users, as proposed in [33] to achieve a rigorous evaluation of identification approaches by accounting for the variation of the performance of our approach depending on the specific group of people.

### 4.5. Experiment-6—Comparison with Existing Works

In this experiment, we compare the performance of our approach to that of 3 existing approaches, which we found to be the ones with best performance in the literature in IMU-based user identification. One of the approaches is an approach based on Gated Recurrent Units (GRU), a type of recurrent network [35]. The second approach is an approach based on a Temporal Convolutional Network (TCN) [36], an architecture that is formed by hierarchy of convolutions to abstract the temporal relations. The third approach is DeepConvLSTM [36], an approach that considers an architecture that combines convolutional models with LSTMs to account for the temporal information in the data.

We measure the performance of each of the approaches using our dataset. In all cases, we consider the same basic configuration, and the results are obtained using a 10-fold cross-validation.

## 5. Results and Analysis

### 5.1. Preliminary Experiment Results

**Figure 10:**
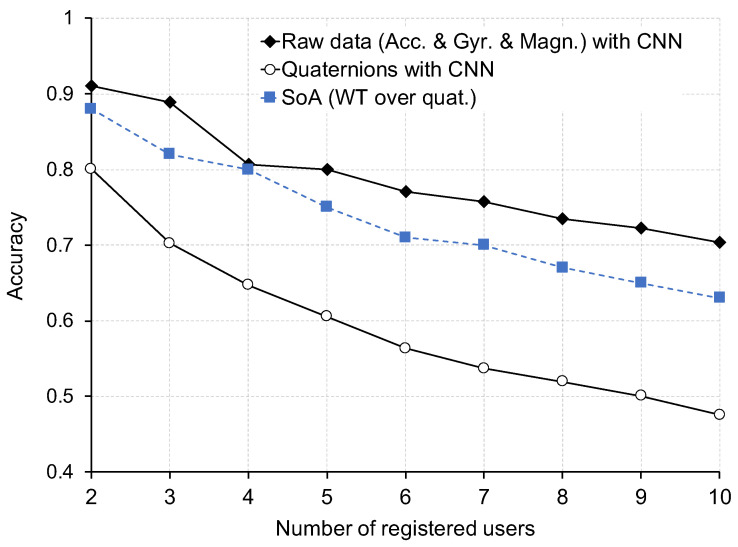
Preliminary experiment results—comparison between CNN with quaternions as input, CNN with raw data (accelerometer (Acc.), gyroscope (Gyr.), and magnetometer (Mgn.), and the state of the art (SoT) in continuous identification, which uses wavelet transform (WT) over quaternions as input and Random Forest (RF) as the ML model.

The results of our preliminary experiment are depicted in Figure 10. The results show that wavelet transform over quaternions, as proposed in [11], clearly outperformed by a considerable margin the case of using directly quaternions instead. However, when considering the raw time-series data directly from the inertial sensors in the IMU, the performance improved slightly with respect to the results obtained using wavelet transform over quaternions. This may be explained by a loss of information due to the transformation. Based on these results, we select, as our baseline, the approach that considers the raw data from accelerometer, gyroscope, and magnetometer, with CNN as the machine-learning model.

### 5.2. Experiment-1 Results—Design Comparison

In Table 1, we present the results of our evaluation for each of the designs we have considered. The best accuracy is achieved by *Design-A-2* with 0.9487, which is not surprising since it is the one that encodes more information. This fact is reflected on the average generation time of this design, which is the longest of all the ones we considered at 0.0538 s. The design that in average takes less time to be generated is *Design-A-3*, taking an average of 0.0109 s. In terms of the best compromise, both in accuracy and generation time, *Design-A-1* seems to be the best option, with an accuracy of 0.9479 and an average generation time of 0.0182 s. Furthermore, as it is B&W, this (together with *Design-A-3*) requires less storage and demands less computation power when training the machine-learning model.

### 5.3. Experiment-2 Results—Voting Criteria

**Figure 11:**
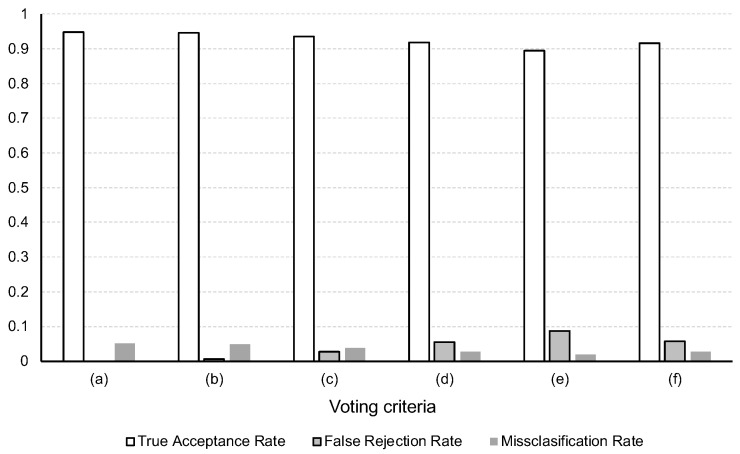
Experiment 2 results—voting criteria comparison: (**a**) First to plurality. (**b**) Plurality. (**c**) Wining by three votes. (**d**) Winning by six votes. (**e**) Winning by nine votes. (**f**) Majority.

In Figure 11, we present the results for Experiment 2, which evaluated our approach for different voting criteria. As can be observed in the figure, the difference in the True Acceptance Rate is not considerable across the different criteria, with a difference of 0.0536 between the best case (i.e., first to plurality with 0.9485) and the worst case (i.e., winning by nine votes with 0.8949).

However, when we consider the False Rejection Rate we can see that the difference between some of the cases is noticeable. These cases, as discussed in Section 4.3.2, may represent an opportunity for a second supporting approach to take the final decision (e.g., direct input from the user), and thus improve the final overall performance of the identification. Thus, for instance, the *winning by 9* criterion has room for a second opinion of nearly 0.1, which could signify as much as 0.99 accuracy using a highly accurate supporting method.

### 5.4. Experiment-3 Results—Training Size

**Figure 12:**
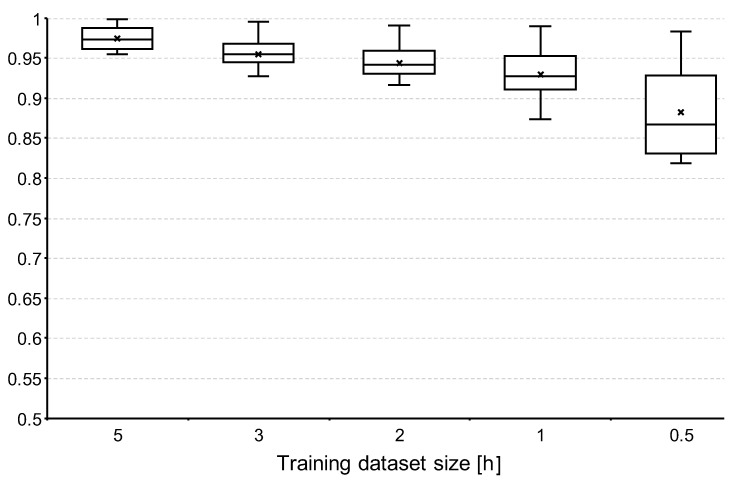
Experiment-3 results—training size.

The results of this experiment are depicted in Figure 12. The average of the maximum values across all training sizes considered is 0.9915. On the other hand, the average of the minimum values across all training sizes considered is 0.8855.

Overall, we can say that the size of the training dataset does have an impact on the performance of our approach, particularly as the number of registered users increases. Specifically, when 5 h of data are considered for training the gap between the best and worst accuracy values is 0.0565, whereas when the training size is reduced to only half an hour, the gap is more than three-times larger at 0.1819.

### 5.5. Experiment-4 Results—CNN Architectures

The results for this experiment on different CNN architectures are presented in Table 2. The specific architecture considered has an impact on the accuracy obtained, however, not in a substantial manner. This demonstrate that our approach is robust to different model architectures.

### 5.6. Experiment-5 Results—Performance for Different Number of Registered Users

**Figure 13:**
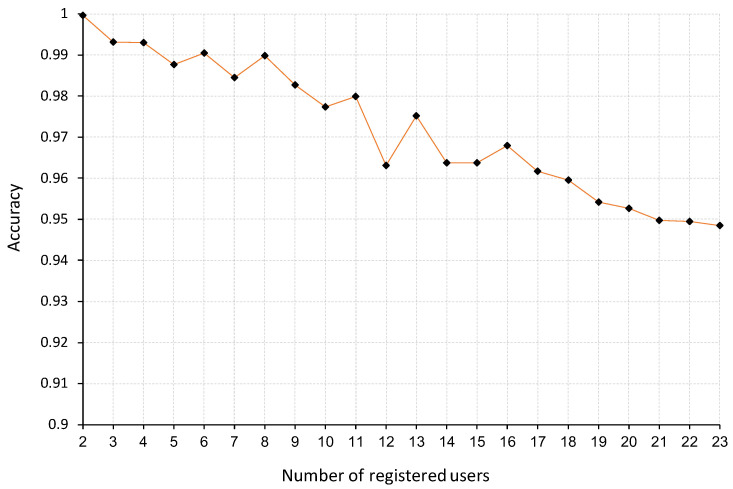
The performance according to the number of registered users.

The results of this experiment are presented in Figure 13. As we can see in the figure, the performance drops as the number of participants increases. Despite this, we observe that the drop in performance is not considerable at slightly more than 5%.

### 5.7. Experiment-6 Results—Comparison with Existing Works

The results of this experiment are presented in Table 3. As we can see, our approach is able to outperform the other approaches. This is despite that our approach makes use of a relatively simple CNN architecture, compared to the more complex architectures employed by the other approaches, particularly DeepConvLSTM, which combines convolutions and recurrent networks.

## 6. Conclusions

In this paper, we proposed an activity-free user-identification approach that built around the idea of using image representations of time-series data recorded by a wrist-worn IMU. Our image representations allowed our method to take advantage of the strengths of convolutional networks regarding image processing tasks and thus improve upon the accuracy and robustness of the state of the art in user identification.

The results from our extensive evaluation provide insights that may be helpful both for practitioners interested in taking our identification method into real-world applications as well as for researchers seeking to further advance the state of the art. We recognize a number of threats to the validity of our evaluation methodology. The first is related to the participants and their characteristics. The number of participants was only 23, which allowed us to evaluate the performance of our approach for certain spaces that are not shared by large groups. At this point, the generalization of our approach to spaces with larger crowds is not guaranteed.

However, our current results for 23 registered users (with an accuracy of 0.9542) show promise for further developing the approach to be able to handle a larger number of registered users. Furthermore, all participants were from a similar age group (25–35 years old). This may have had an impact on the performance of our approach since we focused on motion patterns. One might expect, for instance, the rate of motion of older adults to differ importantly when compared to young adults and children. Therefore, further evaluation including other age groups would be relevant to help support the generalizability of our approach.

Another threat to the validity of our evaluation is the environment considered in the data collection—namely, an office space in a university building. We believe that the office environment that we considered is a space that share similarities with a number of other indoor spaces, and thus our results should be generalizable in that respect. However, in other environments, such as a warehouse, an industrial plant, etc., the dynamics may be quite different. Therefore, a wider range of environments should be considered to further validate the performance and robustness of our approach.

## Figures and Tables

**Table 1:** Experiment-1.1 results—design performance comparison.

Design-ID	Type of Image	Filling Strategy	Avg. Generation Time (s)	Accuracy
Design-A-1	B&W	counter- and clockwise	0.0182	0.9479
Design-A-2	RGB	counter- and clockwise	0.0538	**0.9487**
Design-A-3	B&W	strokes	**0.0109 **	0.9297
Design-A-4	RGB	strokes	0.0227	0.9378
Design-B-1	RGB	counterclockwise	0.0150	0.9325
Design-B-2	RGB	diagonal	0.0155	0.9374
Design-B-3	RGB	strokes	0.0206	0.9423

**Table 2:** Accuracy per CNN architecture.

CNN Architecture			
CNN-0	CNN-1	CNN-2	CNN-3
0.9436	0.9122	0.9485	0.9479

**Table 3:** The performance.

State of the Art		Our Approach	
**Approach**	**Accuracy**	**Accuracy**	**Difference**
DeepSense (GRU) [35]	0.8935		+ 0.0549
TCN [5]	0.9327	0.9485	+ 0.0159
DeepConvLSTM [36]	0.9008		+ 0.0477
